# The effect of maternal common mental disorders on infant undernutrition in Butajira, Ethiopia: The P-MaMiE study

**DOI:** 10.1186/1471-244X-10-32

**Published:** 2010-04-30

**Authors:** Girmay Medhin, Charlotte Hanlon, Michael Dewey, Atalay Alem, Fikru Tesfaye, Zufan Lakew, Bogale Worku, Mesfin Aray, Abdulreshid Abdulahi, Mark Tomlinson, Marcus Hughes, Vikram Patel, Martin Prince

**Affiliations:** 1 Aklilu Lemma Institute of Pathobiology, Addis Ababa University, Addis Ababa, Ethiopia; 2 Department of Psychiatry, Faculty of Medicine, Addis Ababa University, Addis Ababa, Ethiopia; 3 King's College London (Institute of Psychiatry), Health Service and Population Research Department, London, UK; 4 Department of Reproductive Health and Nutrition, School of Public Health, Addis Ababa University, Addis Ababa, Ethiopia; 5 Department of Gynaecology and Obstetrics, Faculty of Medicine, Addis Ababa University, Addis Ababa, Ethiopia; 6 Department of Paediatrics and Child Health, Faculty of Medicine, Addis Ababa University, Addis Ababa, Ethiopia; 7 Department of Psychology, Stellenbosch University, Matieland, South Africa; 8 London School of Hygiene and Tropical Medicine, UK; 9 Sangath, Santemol, Sonarwaddo, Raia, Salcette - Goa - India

## Abstract

**Background:**

Although maternal common mental disorder (CMD) appears to be a risk factor for infant undernutrition in South Asian countries, the position in sub-Saharan Africa (SSA) is unclear

**Methods:**

A population-based cohort of 1065 women, in the third trimester of pregnancy, was identified from the demographic surveillance site (DSS) in Butajira, to investigate the effect of maternal CMD on infant undernutrition in a predominantly rural Ethiopian population. Participants were interviewed at recruitment and at two months post-partum. Maternal CMD was measured using the locally validated Self-Reported Questionnaire (score of ≥ six indicating high levels of CMD). Infant anthropometry was recorded at six and twelve months of age.

**Result:**

The prevalence of CMD was 12% during pregnancy and 5% at the two month postnatal time-point. In bivariate analysis antenatal CMD which had resolved after delivery predicted underweight at twelve months (OR = 1.71; 95% CI: 1.05, 2.50). There were no other statistically significant differences in the prevalence of underweight or stunted infants in mothers with high levels of CMD compared to those with low levels. The associations between CMD and infant nutritional status were not significant after adjusting for pre-specified potential confounders.

**Conclusion:**

Our negative finding adds to the inconsistent picture emerging from SSA. The association between CMD and infant undernutrition might be modified by study methodology as well as degree of shared parenting among family members, making it difficult to extrapolate across low- and middle-income countries.

## Background

Infant undernutrition is a well recognised public health problem in low and middle income countries (LAMIC) [1-3], the cause of which extends beyond mere shortage of food [1,4,5]. Maternal common mental disorders (CMD), characterised by significant levels of depressive, anxiety and somatic symptoms, are highly prevalent in LAMIC [5] and recent studies indicate a potential aetiological role in infant undernutrition [6-15]. Infancy is a critical time for the well-being of the newborn which depends largely on the quality and quantity of care received from the primary caregiver, usually the mother. Postnatal CMD can affect the mother's mental and physical availability to the infant and thus compromise parenting quality [16,17]. A meta-analysis of 19 studies conducted in high-income countries found postnatal depression to have a moderate-to-large adverse effect on maternal-infant interaction during infancy [18]. These findings have been replicated in South Africa, with depressed mothers exhibiting less sensitive engagement with their infants [16] resulting in increased insecure attachment in the infants [19]. Maternal CMD might lead to infant undernutrition through a variety of mechanisms [17,20]. When present during pregnancy, maternal CMD has been associated with an elevated risk of low birth weight [21-23], which in turn is associated with infant undernutrition [6,9]. Postnatal CMD may lead to early cessation of breastfeeding [8] or compromised hygienic feeding practices putting the infant at risk of infectious illnesses [24].

Studies from South Asia [6,8,10,11] have consistently found postnatal CMD to be associated with infant undernutrition after adjusting for potential confounders. However, in Latin America findings have been more mixed, with maternal CMD associated with child under-nutrition in a cross-sectional community sample from Brazil [7,15], but not in a clinic-based study from Jamaica [25] or a large population-based sample in Peru [14]. A similarly inconsistent picture is emerging from sub-Saharan Africa [12-14,26]. In clinic-based studies from Nigeria [12] and Malawi [13], maternal postnatal CMD was associated with infant undernutrition; However, in a population-based cross-sectional sample of two to 18 month old children in Ethiopia [14] and a population-based cohort in South Africa [26], no significant associations were noted between maternal CMD and child undernutrition.

Methodological issues may explain some of the conflicting findings across studies. Variation in the age of children at nutritional assessment, homogeneity of study participants across studies, rural versus urban setting, cultural validity of instruments used to ascertain maternal CMD, use of different nutritional indices as outcomes, as well as different scales of measurement (binary or continuous), the frequencies of exposure and outcomes investigated, the timing at which the effect of exposure on the outcome was evaluated, and the quality of study design may all play a part [11,27]. Furthermore, the majority of published studies fail to take into account the potential impact of maternal CMD in pregnancy upon infant under-nutrition, mediated through low birth weight. Studies from LAMIC have tended to show that the prevalence of maternal CMD is higher in pregnancy than in the postnatal CMD, underlining the importance of examining the impact of antenatal CMD. Only one study, from Pakistan, has evaluated the effect of maternal CMD in pregnancy on child nutritional status prospectively using a population based cohort [9] and showed that CMD in pregnancy significantly compromised the nutritional status of infants at six and twelve months of age. In sub-Saharan Africa, health service coverage is generally low [3,28] which means that clinic-based studies are examining a selected population; this may lead to bias, since women who seek help because their child is under-nourished and ailing may be more likely to be psychologically distressed.

We now report results from a population based cohort, the Perinatal Maternal Mental Disorder in Ethiopia (P-MaMiE) study [29], with the aim of answering the following questions. In a predominantly rural population in sub-Saharan Africa, after taking account of known risk factors for undernutrition:

(a) does maternal CMD in pregnancy significantly contribute to infant undernutrition at six and twelve months of age?

(b) does postnatal CMD significantly contribute to infant undernutrition at six and twelve months of age?,

(c) compared to infants whose mothers had no experience of CMD either in pregnancy or the postnatal period, are infants whose mothers had CMD (i) in pregnancy only, resolving after giving birth, (ii) postnatally, but not in pregnancy (incident postnatal), and (iii) persistently from pregnancy to the postnatal period ('persistent perinatal'), at a higher risk of being undernourished at six and twelve months of age?

## Methods

### Study design and population

A population based prospective cohort of pregnant women was established [29] within the framework of the demographic surveillance site (DSS) in Butajira [30] 135 km south of Addis Ababa, the capital city of Ethiopia. Participants were followed-up with their new born up to one year postnatal. Eligibility criteria include (a) pregnancy within their third trimester between July, 2005 and February, 2006, (b) ability to communicate in Amharic, the official language of Ethiopia, (c) being a resident of the DSS site, and (d) consenting to participate in the study. The DSS enumerators identified pregnant women during their routine surveillance. Eligible women were then interviewed by female data collectors employed to work full-time on the P-MaMiE project. Traditionally people in the study area grow maize and "false banana" Ensete (*Ensete ventricosun*) for subsistence and produce chilli-peppers and khat (*Catha edulis*, a natural stimulant) as cash crops. In recent years, however, the population has been affected by periodic food insecurity. There is a primary health service and primary schools for residents within a maximum distance of 5-6 km. Butajira town is the capital of the district within which the DSS is located. It has basic infrastructure including an all-weather road that runs to the bordering districts, a hospital, a health centre, drug stores, electricity, and digital telephone services.

## Measures

### Anthropometric measurements

Growth measurements were taken by project data collectors, DSS enumerators and community health agents (CHAs). In six sub-districts (the smallest government administrative unit) CHAs who lived and worked in the same sub-district were trained to measure birth weight. During recruitment, participating women were requested to inform the CHA immediately after giving birth to enable the neonate to be weighed ideally within 24 to 48 hours of birth. The remaining four sub-districts had no suitable health worker to measure birth weight and that information was not collected. Infant weight, including birth weight, was measured using SECA 725 scales measuring to an accuracy of 10 g. Infant length was obtained using a locally adapted measuring board. First authors (GM and CH) and one of the collaborators (FT) trained all individuals involved in growth measurements to minimise inter-individual variability.

### Mental health measure

CMD was measured during the third trimester of pregnancy and at two months postnatal using the locally validated Self-Reporting Questionnaire (SRQ-20) [31]. The SRQ-20 is composed of twenty yes/no items asking about the experience of depressive, anxiety, panic and somatic symptoms in the preceding 30 days [32]. The SRQ-20 generates a continuously distributed scale score indicating overall psychological morbidity. In the current study area SRQ-20 showed acceptable convergent validity both as a linear scale and as ordered categories of SRQ symptom burden: no symptoms (scored 0), low symptoms (one to five) and high symptoms (six and above)[31]. To address the current objectives, the total score was dichotomised (SRQ-20 < 6 versus SRQ ≥ 6), high scores indicating a high level of CMD. Three different exposure variables of CMD were considered: (1) antenatal CMD - prevalent cases, (2) postnatal CMD - prevalent cases, (3) four level categorical exposure of CMD with the following categories - never had CMD (never exposed), antenatal CMD resolving after birth (antenatal only), incident postnatal CMD (postnatal only), and 'chronic' CMD (high SRQ-20 score antenatally and postnatally)

### Other covariates

Potential confounding variables were grouped into domains as shown below:

(1) *Household characteristics*: residential area (urban or rural), number of children aged under five years, age of husband and three composite scores:

a. Poverty index including the following variables: non-literate wife, non-literate husband, do not own radio, do not own bed, do not possess valuable goods like gold and jewellery, own home, possess large animals, possess small animals, animals spend night within the living room, house has a window. Individual items of this scale were identified through a rigorous process including exploratory and confirmatory factor analysis. The final scale score was obtained by adding individual items with equal weight. The resulting scale had a Cronbach alpha value of 0.73, indicating an acceptable level of internal consistency.

b. Poor sanitary conditions scale including: not having a toilet facility, not having safe water and disposing of rubbish on the field. We aggregated these three variables as all of them are known risk factors of undernutrition in Ethiopia even though the internal consistency of the resulting scale was relatively low: Cronbach alpha = 0.49.

c. Support to the mother, including: able to visit friends, enough help at home, enough help with looking after children, enough help from husband, no experience of violence. The resulting scale had a Cronbach alpha value of 0.47 which is relatively low; however, these items measure quite different sources of support and we would not expect them to correlate highly.

(2) *Child characteristics*: gender, vaccination status at two months of age, history of severe illness before the age of two months and birth weight (low birth weight, normal birth weight and no birth weight available).

(3) *Maternal characteristics: *Age, height, mid upper arm circumference, type of marriage (polygamous versus non-polygamous), substance use (either chewing khat or drinking alcohol at least weekly), at least one obstetric complication during current delivery (prolonged labour (>24 hours) or assisted delivery (normal vaginal delivery versus instrumental/Caesarian section) or self reported post-partum haemorrhage or post-partum fever) and 'autonomy' scale. The degree of household autonomy was assessed by asking whether the participant had to ask her husband before she was able to sell crops (yes/no), spend household money (yes/no), attend women's groups or other meetings(yes/no), purchase medications for herself or her children (yes/no), attend a health facility(yes/no). Responses to the five categories were summed with equal weights resulting in a scale with a Cronbach alpha value of 0.93.

(4) *Early infant feeding practices*: no pre-lacteal feed, given colostrums, initiation of breast-feeding within one hour of delivery.

### Nutritional indices

Standardized z-scores (height-for-age and weight-for-age) were generated using the new WHO reference population [33]. These scores were dichotomised at a cut-off of -2. Infants whose scores fell below the cut-off were labelled as undernourished. While lower values of height-for-age (i.e. stunting) reflects reduced skeletal growth as the result of repeated undernutrition (or long-standing undernutrition) lower values of weight-for-age (i.e. underweight) do not differentiate between chronic and acute undernutrition [34].

### Sample size estimation

We hypothesised that the infants born to women with high levels of CMD (SRQ20 ≥ 6) during their third trimester would have a 1.5 times higher risk of being stunted at six months of age compared to infants of mothers with a low SRQ score. Based on the demographic and health survey data [35] we assumed incidence of stunting to be 26.6%. We also expected a prevalence of 20% of CMD during the third trimester. A sample of 850 pregnant women would result in 170 exposed and 680 non-exposed infants which gives a power of 90% allowing a 5% probability of type I error. In the event during the time span of the study recruitment proved unexpectedly successful and we eventually recruited 1065 women

### Data Management

Data were checked in the field by supervisors and usually double-entered on the same day using Epidata [36]. Women were re-interviewed within one week if data were missing. Ongoing quality checks were performed by the supervisors, CH and GM.

### Ethical considerations

Prior to the first interview the women were informed about the objective of the study. Written, informed consent was obtained in keeping with requirements of the Ethiopia ethics committee. As the majority of women were non-literate, the form was read out and participants were asked to give a finger-print to signify willingness to participate. Arrangements were made within locally existing public health institutions for the study project to pay all health-related expenses of the women and children participating in the study. The study was granted ethical approval from the National Ethics Review Committee in Ethiopia and the Research Ethics Committee of King's College London in the UK.

### Data Analysis

Data analysis was restricted to singleton infants who had growth measurements at six or twelve month follow-up. Means and proportions were used to describe continuous and categorical characteristics, respectively. Independent sample t-tests were used to compare mean score of nutritional indices of infants born to mothers with and without a high level of CMD. The proportions of undernourished infants among those born to mothers with and without high levels of CMD were compared using Fisher's exact test. The independent effect of CMD on infant nutritional status was evaluated by defining three main exposure variables: (a) antenatal prevalent case, (b) postnatal prevalent case, and (c) four level categorical exposure variable ("no exposure at both time points" (reference), only antenatal exposure, incident postnatal, and "chronic" or persistent exposure) of CMD. Taking each of the three CMD exposures in turn, the association with infant nutritional status was investigated with logistic regression for binary outcomes (undernourished versus well-nourished) and linear regression for continuous outcomes (weight-for-age and height-for-age z scores). In the process of modelling each outcome (weight-for-age and height-for-age) at each time point (six month and twelve month) three steps were followed: (1) bivariate regression taking one of the three CMD exposure variables, (2) multivariable regression adjusting for the effect of CMD on an outcome for a given domain of covariates (household characteristics, child characteristics, maternal characteristics, or infant feeding practices), (3) multivariable regression fully adjusting the effect of CMD for all covariates. Unadjusted and adjusted odds ratios from logistic regression and unstandardised regression coefficients from linear regression with corresponding 95% confidence intervals were used to assess statistical significance and the magnitude of effects. All data analysis was done using STATA [37] with the probability of type 1 error set at 5%.

## Results

### Cohort characteristics

**Figure 1:**
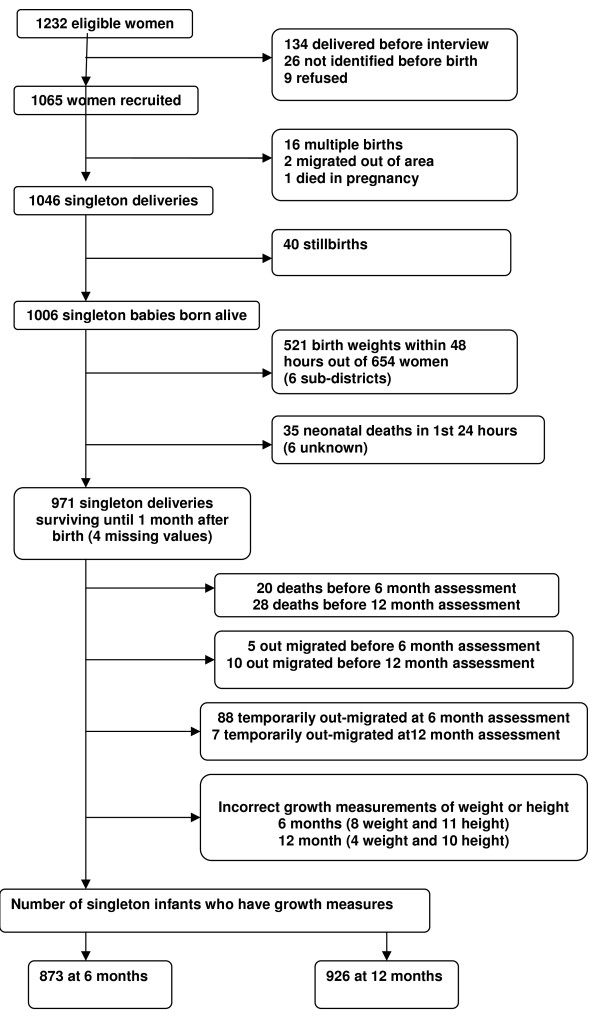
**Follow-up of study participants from screening up to one year postnatal**.

Recruitment and attrition at every stage of follow-up are detailed in Figure 1. One thousand and sixty five (86.3% of eligible) pregnant women were recruited in the third trimester of pregnancy and 128 (12.0%) of them had high levels of antenatal CMD. One thousand and forty-five of the mothers (98.1%) were re-interviewed at two months post partum and 56 (5.4%) of them had postnatal CMD including 26 (2.8%) incident cases. There were 40 stillbirths, 16 multiple births (including one triplet), three losses to follow-up before delivery (one pregnant woman died and two pregnant women out-migrated), and 1006 singleton live births. Anthropometric measurements were available for 873 singletons at six months and for 926 singletons at twelve months of age. The missing cases at six or twelve month did not differ significantly in background characteristics from those included in the present analysis except on the number of under five children and type of marriage. Cases lost to follow-up were less likely to have children under five years old and more likely to be in a polygamous marriage compared to cases whose information is included in this paper.

Selected characteristics of the whole cohort at recruitment are presented in Table 1. Almost all women were married. The large majority belonged to one of three ethnic groups, namely, Meskan (47%), Mareko (14%) and Silti (24%). Most were non-literate (80%), housewives or engaged in farming (88%), and followers of the Islamic religion (78%). The average age of participating women was 27 (sd = 6.4) years and that of their husbands was 36 (sd = 9.2) years. The majority of women in this predominantly rural community had access to safe water (70%) and toilet facilities (63%) but only 22% of women reported safe disposal of rubbish.

**Table 1:** Socio-demographic characteristics and sanitary conditions of the P-MaMiE cohort at baseline, and the rate of follow-up at six and twelve months from the date of birth

*Characteristics*		*6 month follow-up*		*12 month follow-up*	
	
	Baseline sample n(%) or Mean(SD)	Number	Percent or mean(SD)	Number	Percent or mean(SD)
Religion					
Muslim	824(77.6)	673	77.2	722	78.0
Orthodox Christian	161(15.1)	133	15.3	139	15.0
Protestant	66(6.2)	56	6.4	55	5.9
Catholic	12(1.1)	10	1.2	10	1.1
Ethnicity					
Meskan	485(44.5)	404	46.3	436	47.1
Mareko	147(13.8)	119	13.7	123	13.3
Silti	257(24.1)	199	22.8	218	23.5
Sodo	85(8.0)	69	7.9	68	7.3
Others	91(9.6)	81	9.3	81	8.8
Currently married	1055(99.1)	824	99.1	867	99.0
Occupation					
Housewife or farming	933(87.9)	758	87.1	805	87.1
Self or paid employee	129(12.2)	112	12.9	119	12.9
Maternal age in years (n = 1065)	26.9(6.4)	872	26.9(6.2)	926	26.9(6.2)
Educational status of mother					
Formal education	219(20.6)	173	19.8	185	20.0
No formal education	846(79.4)	699	80.2	741	80.0
Age of husband in years (n = 1050)	36.2(9.2)	858	36.0(8.3)	911	36.1(8.9)
Educational status of husband					
Able to read	726(68.6)	594	68.5	632	68.7
Unable to read	333(31.4)	273	31.5	288	31.3
Main source of water					
Protected supply	752(70.8)	600	69.0	644	69.7
Unprotected supply	310(29.2)	270	31.0	280	30.3
Sanitary condition					
Have toilet facilities	674(63.3)	552	63.3	582	62.9
No proper toilet facilities	391(36.7)	320	36.7	344	37.2
Rubbish disposal					
Buries, burns or others	238(22.4)	192	22.0	200	21.6
Disposes on field	826(77.6)	679	78.0	725	78.4

A descriptive summary of infant nutritional status (standardised weight and height/stunting and underweight) stratified by infant age and level of CMD is presented in Table 2. CMD was not significantly associated with infant underweight and stunting at either six or twelve months of age, whether the level of CMD was measured during pregnancy, at two months postnatally, or according to the course of CMD across these two time points. The mean weight-for-age and, height-for-age z scores were lower than those for the WHO child growth standards over the whole year of infancy, independent of CMD. Again, there was no evidence for a statistically significant association between CMD and infant undernutrition assessed using these standardised scores at either six or twelve months.

**Table 2:** Infant nutritional status at the age of six and twelve months stratified by antenatal and postnatal maternal CMD

*Scale of outcome*	*Timing and level of CMD SRQ > = 6 indicating higher level of morbidity*			*Six month time point*		*One year time point*	
		
***Nutritional status as binary outcome***				**Underweight Number (%)**	**Stunting Number(%)**	**Underweight Number(%)**	**Stunting number(%)**
		
	***Pregnancy***						
	Low SRQ score			161(21.0)	205(26.9)	167(20.5)	386(47.8)
	High SRQ score			27(27.6)	25(25.0)	28(25.7)	55(50.9)
	P-value*			0.15	0.72	0.21	0.54
	***Two month postnatal***						
	Low SRQ score			182(22.0)	220(26.8)	188(21.4)	421(48.3)
	High SRQ score			6(15.8)	10(25.6)	6(14.3)	18(43.9)
	p-value*			0.43	1.00	0.34	0.63
	***Pregnancy or postnatal***						
	Low SRQ at all time point			160(21.3)	201(26.9)	162(20.5)	375(47.7)
	High SRQ score at both time points			5(21.7)	6(25.0)	2(8.3)	9(39.1)
	High SRQ score at Postnatal only			1(6.7)	4(26.7)	4(22.2)	9(50.0)
	High SRQ score at Pregnancy only			22(29.3)	19(25.0)	26(30.6)	46(54.1)
	p-value*			0.22	0.99	0.07	0.56

**Nutritional status as continuous outcome**							

				**Weight-for-age Z score Mean(SE)**	**Height-for-age Z-score Mean(SE)**	**Weight-for-age Z score Mean(SE)**	**Height-for-age Z-score Mean(SE)**
		
	***Pregnancy***						
	Low SRQ score			-1.08(0.05)	-1.07(0.06)	-1.05(0.04)	-2.03(0.05)
	High SRQ score			-1.20(0.14)	-1.17(0.14)	-1.16(0.14)	-2.08(0.17)
	P-value*			0.38	0.55	0.37	0.74
	***Two month postnatal***						
	Low SRQ score			-1.10(0.04)	-1.08(0.06)	-1.07(0.04)	-2.03(0.05)
	High SRQ score			-0.84(0.18)	-1.15(0.25)	-0.93(0.15)	-2.15(0.24)
	p-value*			0.21	0.79	0.50	0.63
	***Pregnancy or postnatal***						
	Low SRQ at all time point			-1.11(0.05)	-1.10(0.06)	-1.06(0.05)	-2.05(0.05)
	High SRQ score at both time points			-1.28(0.16)	-1.17(0.15)	-1.32(0.16)	-2.10(0.19)
	High SRQ score at Postnatal only			-0.70(0.20)	-1.11(0.41)	-1.27(0.19)	-2.25(0.27)
	High SRQ score at Pregnancy only			-0.93(0.27)	-1.18(0.31)	-0.67(0.20)	-2.07(0.38)
	p-value*			0.95	0.92	0.12	0.92

Odds ratios and corresponding 95% confidence intervals from bivariate and multivariable logistic regressions assessing the association between the course of CMD from pregnancy to two months postnatally (a four level categorical variable) and infant undernutrition are presented in Table 3. The reference category for this exposure was those mothers who had low levels of CMD at both assessment points. Prior to adjustment for possible confounding factors, infants whose mothers had high levels of CMD during pregnancy which resolved after delivery were more likely to be underweight at 12 months of age (OR = 1.71; 95% CI: 1.05 - 2.80), with a non-significant trend in the same direction at six months (OR = 1.53; 95% CI: 0.91 - 2.60) and for stunting at 12 months (OR = 1.30, 95% CI: 0.83 - 2.03). The excess risk for an infant being underweight at 12 months of age remained significant after adjusting for infant characteristics and early infant feeding practices of the mother but became statistically non-significant after adjusting for maternal characteristics or household characteristics. Although the risk for underweight at six months and for stunting at twelve months was not statistically significantly associated with antenatal CMD which resolved after delivery, a consistent trend in the same direction still remained after adjusting for each group of confounding variables. In the final multivariable model, adjusting for all of the potential confounders simultaneously, the course of CMD was not significantly associated with infant nutritional status at either six or twelve months of age.

**Table 3:** Unadjusted, partially adjusted and fully adjusted effect of antenatal only, incident postnatal and chronic CMD on infant undernutrition at the age of six and twelve months in the P-MaMiE study

*Model*	Timing for main exposure	*Six month time point*		*One year time point*	
		
		Underweight OR(95% CI)	Stunting OR(95% CI)	Underweight OR(95% CI)	Stunting OR(95% CI)
Unadjusted					
	Never exposed	1	1	1	1
	Pregnancy only	1.53(0.91, 2.60)	0.90(0.52, 1.56)	1.71(1.05, 2.80)	1.30(0.83, 2.03)
	Postnatal only	0.26(0.03, 2.02)	0.99(0.31, 3.13)	1.11(0.36, 3.42)	1.10(0.43, 2.80)
	Both time points	1.03(0.38, 2.81)	0.90(0.35, 2.31)	0.35(0.08, 1.52)	0.71(0.30, 1.65)
Adjusted for Household characteristics					
	Never exposed	1	1	1	1
	Pregnancy only	1.44(0.82, 2.54)	0.90(0.51, 1.58)	1.46(0.86, 2.47)	1.12(0.70, 1.79)
	Postnatal only	0.33(0.04, 2.57)	0.97(0.30, 3.09)	1.31(0.41, 4.17)	1.26(0.28, 3.26)
	Both time points	1.09(0.40, 3.03)	0.89(0.35, 2.29)	0.32(0.07, 1.40)	0.68(0.29, 1.62)
Maternal characteristics					
	Never exposed	1	1	1	1
	Pregnancy only	1.65(0.95, 2.87)	0.84(0.47, 1.50)	1.54(0.91, 2.63)	1.27(0.79, 2.04)
	Postnatal only	0.23(0.03, 1.78)	0.87(0.27, 2.78)	0.99(0.32, 3.13)	1.04(0.40, 2.26)
	Both time points	0.98(0.35, 2.73)	0.82(0.32, 2.14)	0.30(0.07, 1.30)	0.69(0.29, 1.63)
Infant characteristics					
	Never exposed	1	1	1	1
	Pregnancy only	1.68(0.97, 2.91)	0.98(0.56, 1.71)	1.75(1.05, 2.94)	1.39(0.87, 2.21)
	Postnatal only	0.30(0.04, 2.34)	0.98(0.31, 3.14)	1.17(0.38, 3.61)	1.08(0.42, 2.75)
	Both time points	0.98(0.36, 2.70)	0.87(0.34, 2.23)	0.35(0.08, 1.49)	0.70(0.30, 1.64)
Feeding practices					
	Never exposed	1	1	1	1
	Pregnancy only	1.68 (0.97, 2.91)	0.98(0.56, 1.71)	1.75(1.05, 2.93)	1.39(0.87, 2.21)
	Postnatal only	0.30(0.04, 2.34)	0.98(0.31, 3.14)	1.17(0.38, 3.61)	1.08(0.42, 2.75)
	Both time points	0.98(0.36, 2.70)	0.87(0.34, 2.23)	0.35(0.08, 149)	0.70(0.30, 1.64)
Fully adjusted					
	Never exposed	1	1	1	1
	Pregnancy only	1.43(0.76, 2.71)	0.86(0.46, 1.62)	1.07(0.58, 1.97)	1.14(0.67, 1.96)
	Postnatal only	0.21(0.02, 1.86)	0.67(0.20, 2.27)	1.07(0.33, 3.62)	1.06(0.39, 2.94)
	Both time points	0.85(0.29, 2.50)	0.64(0.24, 1.73)	0.25(0.06, 1.15)	0.66(0.27, 1.63)

**From Literature**					
Rahman et al 2004**	Chronic cases	5.9(2.7, 12.8)	5.5(1.9, 16.0)	3.5(2.2, 5.6)	3.2(1.9, 5.6)

** Measures of association reported by the authors are unadjusted estimates of relative risks

The results of bivariate and multivariable logistic regression with antenatal CMD (prevalent cases) and postnatal CMD (prevalent cases) as the main exposures for infant undernutrition are presented in Table 4. There was no significant effect of either antenatal or postnatal CMD upon infant nutritional status at either time point, both before and after adjusting for potential confounding variables. In a multivariable logistic regression use of SRQ score as a continuous exposure variable did not altered our finding of no association between CMD and infant undernutrition.

**Table 4:** unadjusted, partially adjusted and fully adjusted effect of antenatal and postnatal prevalent CMD on infant undernutrition at the age of six and twelve months in the P-MaMiE study

*Model*	Timing for main exposure	*Six month time point*		*One year time point*	
		
		Underweight OR(95% CI)	Stunting OR(95% CI)	Underweight OR(95% CI)	Stunting OR(95% CI)
Unadjusted					
	Antenatal	1.43(0.89,2.30)	0.91(0.56, 1.46)	1.34(0.84, 2.12)	1.13(0.76, 1.70)
	Postnatal	0.66(0.27, 1.61)	0.94(0.45, 1.97)	0.61(0.25, 1.47)	0.84(0.45, 1.58)
Adjusted for					
Household characteristics					
	Antenatal	1.37(0.83, 2.27)	0.90(0.55, 1.47)	1.13(0.69, 1.84)	1.00(0.65, 1.52)
	Postnatal	0.76(0.31, 1.87)	0.93(0.44, 1.95)	0.63(0.26, 1.54)	0.88(0.46, 1.68)
Maternal characteristics					
	Antenatal	1.50(0.91, 2.48)	0.84(0.50, 1.39)	1.17(0.71, 1.92)	1.10(0.72, 1.69)
	Postnatal	0.60(0.24, 1.47)	0.85(0.40, 1.80)	0.53(0.22, 1.29)	0.80(0.42, 1.52)
Infant characteristics					
	Antenatal	1.50(0.92, 2.46)	0.96(0.59, 1.56)	1.33(0.82, 2.14)	1.18(0.78, 1.79)
	Postnatal	0.68(0.28, 1.65)	0.91(0.44, 1.91)	0.62(0.26, 1.49)	0.82(0.44, 1.55)
Feeding practices					
	Antenatal	1.52(0.93, 2.49)	0.96(0.59, 1.57)	1.32(0.82, 2.13)	1.18(0.78, 1.78)
	Postnatal	0.67(0.28, 1.65)	0.92(0.44, 1.92)	0.61(0.25, 1.47)	0.81(0.43, 1.53)
Fully Adjusted					
	Antenatal	1.28(0.73, 2.24)	0.80(0.46, 1.38)	0.81(0.46, 1.43)	1.00(0.62, 1.60)
	Postnatal	0.56(0.22, 1.46)	0.66(0.30, 1.45)	0.52(0.21, 1.32)	0.80(0.40, 1.59)

**Available Evidence from relevant Literature**					

Rahman et al 2004	Antenatal	3.5(1.5, 8.6)	3.2(1.1, 9.9)	3.0(1.5, 6.0)	2.8(1.3, 6.1)
Adewuya et al 2008	Postnatal	4.21(1.34, 13.20)	3.34(1.18, 9.55)	-------	-------
Adewuya et al 2008^§^	Postnatal	3.19(1.21, 8.40)	3.21(1.03 10.47)	-----	-----
Patel et al 2003 **	Postnatal	Varies between 2.5 and 3.5	Varies between 3.2 and 3.6	-------	-------
Rahman et al 2004**	Postnatal	--------	-------	2.8(1.2, 6.8)	-------
Anoop et al 2004***	Postnatal	--------	-------	3.1(0.9, 9.7)	--------
Tomlinson et al 2006+	Postnatal	0.25(0.03, 2.09)	1.78(0.69, 4.63)		
Tomlinson et al 2006++	Postnatal			2.32(0.90, 6.00)	2.52(0.98, 6.47)
Tomlinson et al 2006+++	Postnatal			1.10(0.27, 4.46)	2.44(0.70, 8.58)
Harpman etal 2005 (ET)	Postnatal			1.1(0.9, 1.3)	0.9(0.7, 1.1)
Harpman etal 2005 (PE)	Postnatal			0.8(0.6, 1.1)	1.1(0.9, 1.4)
Harpman etal 2005 (VE)	Postnatal			1.3(1.0, 1.7)	1.2(0.9, 1.7)
Harpman etal 2005 (IN)	postnatal			1.1(0.9, 1.3)	1.4(1.2, 1.6)
Surkan et al 2008^§§^	postnatal			0.6(0.2, 1.7)	1.8(1.1, 2.8)

* the models are not fully adjusted and hence no single estimate for adjusted odds ratio

** The result is for nine month rather than for 12 months of infant age

*** The result is at a median infant age of 10.5 month rather than 12 months of age

+ unadjusted effect of concurrent CMD at two month time point

++ unadjusted effect of two month postnatal CMD on 18 month nutritional status

+++ unadjusted effect of concurrent CMD at 18 month of age

^§ ^unadjusted effect of postnatal CMD on three month nutritional status

§§ Reported effects are on nutritional status of 6-24 year old children

ET = Ethiopia; PE = Peru, VE = Vetnam; IN = India

Modelling of the association between CMD and infant nutritional status was repeated using linear regression For this purpose, weight and length of infants in standard deviation units were kept as continuous outcome variables and CMD as the main exposure was defined as in the methods section (antenatal - prevalent, postnatal - prevalent and four level exposure - never/antenatal only/incident postnatal/chronic). None of the findings showed statistically significant effect of CMD on nutritional status of infants (result not shown). Use of the SRQ score as a continuous exposure variable did not alter our finding of no association between CMD and height-for-age z score or weight-for-age z score, either at six months and at twelve months of age.

## Discussion

In this population-based prospective study from rural Ethiopia we evaluated the effect of maternal CMD in pregnancy and at two months postnatal upon infant nutritional status assessed at six and twelve months of age. The prevalence of infant undernutrition, indicated by stunting (length for age z score less than -2) and being underweight (weight for age z score less than -2), was high at both time points; however, the prevalence of maternal CMD was relatively low, particularly at the two month postnatal time-point. In fully adjusted multivariable analyses, infant exposure to maternal CMD in pregnancy, at two months postnatal, or at both perinatal time-points was not significantly associated with infant nutritional status at six months or at one year of age. When maternal CMD was considered as a four level categorical variable (never, pregnancy only, incident postnatal only, persistent perinatal) CMD in pregnancy that resolved following delivery was associated with the infant being underweight at one year. However, this association became non-significant after adjusting for household and maternal characteristics. Neither this nor any other effects of maternal CMD were significant in the fully adjusted model, whether we considered nutritional indices as dichotomous or as continuous outcomes.

The credibility of the current results is based on the strengths of the study which include: (a) a large population-based sample from an area with a high prevalence of infant undernutrition and low levels of loss to follow-up over 12 months, (b) the first study from sub-Saharan Africa and the second from a LAMIC setting to ascertain CMD during pregnancy as well as at two months postnatally and to assess their effects on infant outcomes prospectively, (c) assessment of infant nutritional status at both six and twelve months of age, and (d) adjustment for a large number of potentially confounding variables. However, the study has some limitations. The SRQ-20 is a scale-based measure of maternal CMD symptoms, rather than providing a definitive diagnostic assessment of mental disorder. In three[6,9,12] out of the four [26] studies that made use of standardised clinical diagnostic measures of maternal depression, a positive association with infant undernutrition was detected. That said, the SRQ-20 has been used extensively in the study area for assessment of CMD in the general population [38] and was validated before the current study on pregnant and postnatal women from the same geographical area [31]. Nevertheless, the assessment of CMD in this setting is by no means straightforward [31] and misclassification of cases is likely to have biased any genuine association towards the null. The low prevalence of maternal CMD that we observed postnatally would also have reduced the study power to detect an effect on infant undernutrition, potentially leading to type II error.

The possible association between maternal CMD and child undernutrition in LAMIC has captured the attention of researchers in recent years, and has been tested using epidemiological studies of varying methodological quality that may have contributed to the different findings across settings. However, consistent and significant associations have been observed in south Asia independent of these and other heterogeneities.

The two previously published population-based cohort studies [9,26], both using diagnostic measures of maternal depression, present conflicting results: in periurban South Africa no association was found with any index of child nutritional status at 18 months [26], whereas in rural Pakistan [9] the association was seen with categorical indicators of under-nutrition at both six and 12 months (underweight: OR = 3.5; 95% CI: 1.5 - 8.6 at six months and OR = 3.0; 95% CI: 1.5 - 6.0 at 12 months, and stunted: OR = 3.2; 95% CI: 1.1 - 9.9 at six months; OR = 2.8; 95% CI: 1.3 - 6.1 at 12 months). Our study sample is most comparable to the Pakistan study, although socioeconomic measures indicate greater poverty in the Ethiopia sample, for example, substantially lower levels of household electricity and flush toilets compared to Pakistan[9]. It is possible that the level of poverty in our study sample might have overwhelmed other factors, such as maternal CMD, affecting the nutritional status of the infant[29]. Outside of South Asia, most of the negative findings from South America [14,39] and sub-Saharan Africa [14,26] originated from population-based studies, while most of the positive findings [7,12,13] are from clinic-based studies. The nature of the selection bias is not immediately evident, but the potential is clearly present given the limited access and use of routine antenatal and obstetric care, particularly in sub-Saharan Africa.

The timing of measurement of infant undernutrition could have relevance, with the two previously negative studies from sub-Saharan Africa evaluating children at an older age: 18 months[26] and 6 to 18 months (>50% over 12 months of age)[14]. Similarly for the negative study from Jamaica (9 to 30 months) [25]. In the Nigeria study, a significant association between postnatal CMD and infant undernutrition was only found at three and six, but not at nine months of age[12]. Although the Bangladesh study found the reverse, that maternal CMD was only associated with infant undernutrition at 12 months and not at six months, this is likely to have occurred because maternal CMD was measured at 12 months and thus showed a stronger association concurrently[11]. However, in our Ethiopia study, no association with infant undernutrition was apparent at either six or 12 months of age.

Most previous studies reported categorical indicators of infant nutritional status. Where the analyses were repeated for both categorical and continuous nutritional indices, only the categorical measure was associated with maternal CMD in Brazil[15,39], and neither were associated in South Africa[26], the latter in keeping with our study. When the two have been compared in the same study, impaired linear growth (length-for-age; stunting) has more often been associated with maternal CMD than the composite nutritional indicator of weight-for-age[11,13,39]. In our study we used both length-for-age and weight-for-age as nutritional indicators, and neither was associated with maternal CMD.

Although our study adjusted for a broader range of potential confounding variables than most other studies, there is little evidence that over-adjustment occurred as no positive associations were observed in the univariate analyses.

Persistent perinatal CMD could impact on nutrition during pregnancy and after birth. The nature of any interaction between CMD in pregnancy and the postnatal period to cause under-nutrition is unclear. Contrary to the current findings there is strong evidence in Pakistan [9] showing that chronic perinatal CMD significantly increases the risk of infant underweight (relative risk (RR) = 5.9; 95% CI: 2.7 to 12.8 at six months, RR = 3.5; 95% CI: 2.2 to 5.6 at 12 months) and stunting (RR = 5.5; 95% CI: 1.9 to 16.0 at 6 months and RR = 3.2; 95% CI: 1.9 to 5.4 at 12 months). However, in the Pakistan study there was little remission of depression in pregnancy, or incidence of postnatal depression. A very low prevalence of persistent CMD in the current study compromised the power to detect any meaningful effect.

Including the current study, five other studies, from Ethiopia [14], South Africa [26], Brazil [39], Peru [14] and Jamaica [25] have failed to replicate the association between maternal CMD and infant undernutrition seen in South Asia[6,8-11,14]. We have previously found that maternal CMD in pregnancy in this Ethiopian cohort was not associated with low birth weight[29], again in contrast to the findings from South Asia. The true absence of an adverse effect of maternal CMD in pregnancy or the postnatal period on child nutritional status in Ethiopia is thus possible. When interpreting their negative findings from Ethiopia and Peru compared to India and Vietnam, Harpham et al. call for qualitative exploration for the reasons for such differences and speculate that the 'pressurised cultural role of women in relation to childcare' in South Asia might be exacerbated by a child who is failing to thrive, leading to worsening maternal mental health[14]. In Ethiopia, shared parenting practices within families and neighbourhoods may have diluted any negative effect of postnatal and persistent CMD. Informal feedback from our project data collectors suggests that children in this community are considered as potential future capital, giving higher parity mothers an elevated social rank compared to mothers of the same age with a smaller number of children. One of the common reasons to justify polygamous marriage in the community is the demand for more children by the husband. This could mean that maternal CMD becomes less prevalent and/or severe as the family expands, and the presence of more siblings for the child also facilitates shared parenting. However, at this stage there are no clear answers for why maternal mental disturbance appears to have such a significant effect on child growth in some countries and not in other countries, including Ethiopia.

## Conclusions

Our population-based study from rural Ethiopia found no significant association between maternal perinatal CMD (i.e. during the third trimester of pregnancy, two months postnatal and persistently from pregnancy up to two month postnatal) and infant undernutrition at six or twelve months of age. This result, in the context of other research, questions the universality of the proposed causal link between CMD and impaired infant growth across LAMIC.

## Competing interests

The authors declare that they have no competing interests.

## Authors' contributions

CH, MP, AA conceived the idea and CH designed the study. GM and CH developed data collection instruments, coordinated data collection and have full access to the raw data. GM analysed the data and drafted the manuscript. CH, MD, and MP contributed to data analysis. CH, MD, MP, VP, MH, MT, FT critically commented on the draft manuscript. All authors contributed to and have approved the final manuscript.

## Pre-publication history

The pre-publication history for this paper can be accessed here:

http://www.biomedcentral.com/1471-244X/10/32/prepub
